# Characterization of Sodium Channel Mutations in the Dengue Vector Mosquitoes *Aedes* *aegypti* and *Aedes* *albopictus* within the Context of Ongoing *Wolbachia* Releases in Kuala Lumpur, Malaysia

**DOI:** 10.3390/insects11080529

**Published:** 2020-08-13

**Authors:** Noor Afizah Ahmad, Nancy M. Endersby-Harshman, Nur Ruqqayah Mohd Mazni, Nur Zatil Aqmar Mohd Zabari, Siti Nor Syazwani Amran, Muhammad Kamarul Ridhuan Ghazali, Mohd Arif Abdul Karim, Yoon Ling Cheong, Steven P. Sinkins, Nazni Wasi Ahmad, Ary A. Hoffmann

**Affiliations:** 1 Medical Entomology Unit, Institute for Medical Research, Ministry of Health, Jalan Pahang, Kuala Lumpur 50588, Malaysia; ruqqayahmazni@gmail.com (N.R.M.M.); zatilaqmar27@gmail.com (N.Z.A.M.Z.); norsyazwaniamran@gmail.com (S.N.S.A.); kamarulridhuan88@gmail.com (M.K.R.G.); mohdarif.pps@gmail.com (M.A.A.K.); cheongyl@moh.gov.my (Y.L.C.); nazni@moh.gov.my (N.W.A.); 2 PEARG, School of BioSciences, Bio21 Institute, The University of Melbourne, 30 Flemington Rd, Parkville, Victoria 3010, Australia; ary@unimelb.edu.au; 3 Institute of Infection, Immunity and Inflammation, MRC-University of Glasgow Centre for Virus Research, 464 Bearsden Road, Glasgow G61 1QH, UK; Steven.Sinkins@glasgow.ac.uk

**Keywords:** pyrethroid resistance, target site, *kdr*

## Abstract

**Simple Summary:**

Mosquitoes, *Aedes aegypti* and *Ae. albopictus* are vectors of dengue and must be controlled to prevent and contain outbreaks of this disease. Control by insecticide application is common and pyrethroid insecticides provide rapid knockdown of mosquitoes combined with relatively low mammalian toxicity. However, resistance to pyrethroids and other chemicals is causing problems for mosquito control around the world. In Malaysia, an alternative method of dengue reduction is employed which comprises releases of *Ae. aegypti* mosquitoes infected with a bacterium, *Wolbachia*, found naturally in other insects. *Wolbachia* turns the mosquitoes into incompetent vectors so they do not transmit the disease. *Wolbachia* mosquitoes are reared in the laboratory before release and must be able to survive in the field where they will encounter insecticides. Our study demonstrates benefits of crossing laboratory mosquitoes to those from the field over generations, so that the mosquito lines acquire field resistance characteristics (mutations in the sodium channel gene). We demonstrate that resistance mutations provide a survival advantage to *Wolbachia Ae. aegypti* mosquitoes, which must be maintained in laboratory lines by regular backcrossing. We also describe appearance of a sodium channel mutation in Malaysian *Ae. albopictus* which may indicate that pyrethroid resistance is increasing in this species.

**Abstract:**

Specific sodium channel gene mutations confer target site resistance to pyrethroid insecticides in mosquitoes and other insects. In *Aedes* mosquito species, multiple mutations that contribute to resistance vary in their importance around the world. Here, we characterize voltage sensitive sodium channel (*Vssc*) mutations in populations of *Aedes* *aegypti* from Kuala Lumpur, Malaysia, and look at their persistence in populations affected by ongoing *Wolbachia* releases (a dengue control measure). We also describe a *Vssc* mutation in *Aedes* *albopictus* (F1534L) found for the first time in Malaysia. We show that there are three predominant *Vssc* haplotypes in *Aedes* *aegypti* in this region, which all persist with regular backcrossing, thereby maintaining the original genetic composition of the populations. We identify changes in genotype frequency in closed populations of *Ae*. *aegypti* maintained for multiple generations in laboratory culture, suggesting different fitness costs associated with the genotypes, some of which may be associated with the sex of the mosquito. Following population replacement of *Ae*. *aegypti* by *Wolbachia* in the target area, however, we find that the *Vssc* mutations have persisted at pre-release levels. Mosquitoes in two genotype classes demonstrate a type I pyrethroid resistance advantage over wildtype mosquitoes when exposed to 0.25% permethrin. This resistance advantage is even more pronounced with a type II pyrethroid, deltamethrin (0.03%). The results point to the importance of these mutations in pyrethroid resistance in mosquito populations and the need for regular backcrossing with male mosquitoes from the field to maintain similarity of genetic background and population integrity during *Wolbachia* releases.

## 1. Introduction

Dengue is a mosquito-borne infection that has become a major international public health concern. Malaysia is one of the most affected countries, with a total of 130,101 dengue cases and 182 deaths being recorded for 2019. This reflects an increase of 61.4% in terms of dengue cases and 23.8% in terms of deaths as compared to a similar period in 2018 [1]. In Malaysia, reducing human cases of dengue still relies on the control of vector mosquitoes, through fogging using permethrin, deltamethrin and malathion to kill adult mosquitoes, and larviciding using temephos to kill larvae. However, problems with insecticide resistance have arisen [2,3,4] due to a strong selective pressure from frequent application of insecticides which eventually will threaten the continued success of current vector control interventions.

In Malaysia, selection for insecticide resistance in *Aedes* species likely began with fogging operations using malathion which has continued since the early 1970s, and fogging with permethrin which has occurred since early 1996 [5]. Regular indoor residual spraying with dichlorodiphenyltrichloroethane (DDT) was also undertaken in Malaysia for 26 years, which then switched to deltamethrin in 1997 [6]. Pyrethroid insecticides are still widely used to control mosquito vector populations [7,8], not only by the Ministry of Health, but also by private companies and communities to control mosquitoes as well as other household pests [9]. The versatility of pyrethroids which can be applied in different forms and in a range of situations has made this group the most intensively used insecticide for vector control against dengue in Kuala Lumpur.

Wan Norafikah et al. [3] found permethrin resistance in *Aedes aegypti* from Kuala Lumpur and identified a metabolic resistance component. Ishak et al. [4] found highest levels of pyrethroid resistance in *Ae*. *aegypti* and *Ae*. *albopictus* from Kuala Lumpur when compared to mosquitoes from Penang, Johor Bharu, and Kota Bharu. *Aedes albopictus* remained susceptible to pyrethroid insecticides except in Kuala Lumpur, where it showed moderate resistance to a type I and a type II pyrethroid [4]. The presence of permethrin (type I pyrethroid) resistance in field-collected *Ae*. *aegypti* from several urban areas in Kuala Lumpur has been reported, with the resistance ratio ranging from 4.28 to 4.47-fold, whilst for larvae, the resistance ratio ranged from 5.29 to 5.57-fold (commercial grade, 10.9% a. i. *w*/*v*) [3]. Loke et al. [2] reported temporal variation in susceptibility to insecticides in a dengue endemic area in Shah Alam, with organophosphates being the most toxic to *Ae. aegypti* among the four classes of insecticides tested overall.

Pyrethroid resistance is a major concern in Malaysia and two mechanisms of resistance (target-site resistance (also known as knockdown resistance, *kdr*) and metabolic resistance) have been identified in *Ae*. *aegypti* [4,10,11], while metabolic detoxification and possibly reduced cuticular penetration have been implicated in resistance in *Ae*. *albopictus* [12]. Although metabolic resistance is an important mechanism of pyrethroid resistance in *Ae*. *aegypti* in Malaysia, target-site resistance involving the voltage-sensitive sodium channel (*Vssc*) appears particularly widespread. Two mutations, V1016G and F1534C, have functional effects on the sodium channel [13] and a third, S989P, may enhance resistance if found in conjunction with the V1016G mutation [14]. In southeast Asia, the frequencies of resistance alleles of these three mutations in *Ae*. *aegypti* vary between sites, but tend to be common. In Indonesia, the V1016G mutation and the associated S989P mutation are more common than F1534C [14,15]. The V1016G mutation is widely distributed and has been detected in *Ae*. *aegypti* from Malaysia, Indonesia, Thailand, Singapore and China [16]. F1534C is common in *Ae*. *aegypti* from Thailand [17], Vietnam [18], and further afield in the Caribbean [19] and Brazil [20]. V1016G is often associated with S989P and, in parts of southeast Asia and throughout the Indo-Pacific region, the 1016G resistant homozygote appears to be tightly linked to the F1534 susceptible homozygote [14,21,22]. The co-occurrence of homozygous mutations of 1016G/1534C/989P within an individual is rare, but has been detected at low frequency in *Ae*. *aegypti* populations in Myanmar [23], Indonesia [15] and Saudi Arabia [24].

The current study aimed to screen samples of *Ae*. *aegypti* and *Ae*. *albopictus* from Kuala Lumpur for sodium channel mutations that can be used as a convenient marker for one mechanism of pyrethroid insecticide resistance in mosquitoes in this location where a *Wolbachia* mosquito population replacement strategy is in progress [25]. In detail, we aimed to:

1. Monitor insecticide resistance in *Aedes aegypti* in the vicinity of *Wolbachia* program release sites in Shah Alam, Selangor (30 km from Kuala Lumpur) and look for changes in the genetic constitution of resistance at those sites.
2. Characterize the genotypes at three loci in the sodium channel for the V1016G, F1534C and S989P mutations and assess the association between the mutations and pyrethroid resistance in *Ae*. *aegypti* in Shah Alam.
3. Assess the utility of backcrossing laboratory colony *Wolbachia* mosquitoes to the field population to maintain consistency in sodium channel mutations and enhance survival of the released mosquitoes.
4. Sequence the region of the sodium channel gene around codon 1534 in *Ae*. *albopictus* (a second vector of dengue which may be the target of future *Wolbachia* release programs in Malaysia) from AU2, Keramat, Selangor (Kuala Lumpur boundary) to look for resistance mutations which have been identified in some populations, though not yet from Malaysia.

The momentum for this work stems from a *Wolbachia* release program being undertaken in Kuala Lumpur where uninfected *Ae*. *aegypti* populations are currently being replaced by *w*AlbB *Wolbachia*-infected mosquitoes [25], which can prevent the transmission of dengue and other arboviruses [26]. A key aspect to the success of *Wolbachia* releases is to ensure that the genetic background of the release strain is similar to that of the target population [27,28], which contributes to production and release of competitive mosquitoes. While *Wolbachia* does not directly affect resistance in *Ae. aegypti* [29], matching insecticide resistance to background levels is a key component of the genetic background given that releases can fail if there is inadequate insecticide resistance, as happened in *w*Mel *Wolbachia* releases in Rio de Janeiro [30,31]. Monitoring of resistance in release stock can be greatly facilitated by using molecular markers and we therefore had the subsidiary aim of maintaining the relevant sodium channel mutations in *Wolbachia*-infected mosquito strains being used for release in Malaysia to ensure that these strains are not disadvantaged relative to resident populations under current patterns of pyrethroid insecticide use.

## 2. Materials and Methods

### 2.1. Sampling and the Wolbachia Release Program 

**Figure 1:**
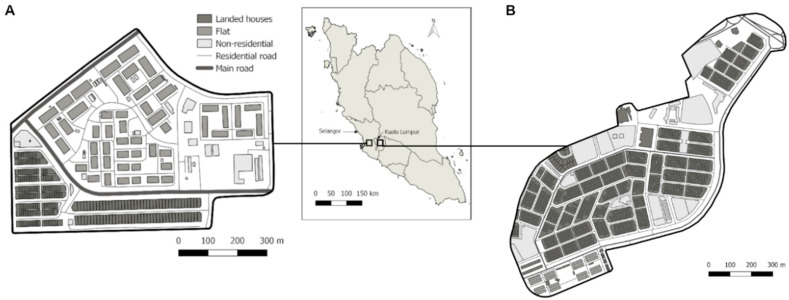
Map of collection sites of *Aedes aegypti* and *Ae*. *albopictus*, (**A**) PKNS, Shah Alam; (**B**) AU2, Keramat.

The sampling sites were PKNS, Shah Alam (area: 0.60 km^2^), a mix of 51 blocks of 5-storey low rise flats and AU2, Keramat, (area: 0.71 km^2^), a housing estate with landed property of terrace houses (Figure 1). 

To collect *Aedes* spp., ovitrapping was conducted following the guidelines of the Ministry of Health on ovitrap deployment [32]. Black plastic containers of 300 mL volume were used as the ovitrap. Hardboard measuring 10 cm × 2.5 cm × 0.3 cm was used as an oviposition paddle and was placed in the ovitrap container to allow mosquitoes to lay eggs on its surface. Clean tap water was added to a level of 5.5 cm [33].

The surveillance at PKNS, Shah Alam was conducted on 14 November 2016, prior to the initiation of *Wolbachia* releases, and post-release on 11 February 2019. A total of 100 ovitraps were placed in covered areas on each occasion. Ovitraps were placed at the staircase and at the corridor of the flats in Shah Alam. In AU2, Keramat, ovitrapping was conducted on 16 May 2017. A total of 130 ovitraps were placed outdoors. After five days of exposure, the ovitraps were recovered and brought back to the Medical Entomology Unit laboratory, at the Institute for Medical Research (IMR) for species identification. Samples of *Ae*. *aegypti* from Shah Alam and *Ae*. *albopictus* from AU2 Keramat were reared for insecticide resistance testing and genotyping.

### 2.2. Colony Samples

Adult male and female *Ae*. *aegypti* were sampled over time from the laboratory colony used for the *Wolbachia* release program, which was maintained at several thousand individuals across generations. Mosquitoes from the colony are almost all infected with the *Wolbachia* strain *w*AlbB [25]. Generations sampled were F3, F8, F9, F13 and F20 reared since the third backcross of *Wolbachia*-infected females to field males (see Nazni et al. [25]). These stocks were designated B3F3 to B3F20, while the colony before backcrossing was designated as B0. A fourth and fifth backcross was conducted to produce stocks with the B5 designation and these were sampled directly after backcrossing (B5F0) and three generations later (B5F3). Mosquitoes from the IMR colony were preserved in absolute ethanol prior to DNA extraction and molecular screening for sodium channel mutations.

### 2.3. Colony Rearing Protocols 

The *w*AlbB infected mosquitoes were maintained at room temperature of ~25 °C with 75 ± 10% relative humidity and a photoperiod of 12:12 h (light/dark). Approximately 500 larvae were reared in a tray filled with 2000 mL of seasoned water (tap water stored overnight to dechlorinate). The immature stages of mosquitoes were supplied with liver powder (Difco^TM^, Becton, Dickinson and Company, Franklin Lakes, NJ, USA) following the regime described by Nazni et al. [25] and adults were supplied with 10% sucrose solution incorporated with liquid B-complex. Adults were blood fed on white mice and allowed to lay eggs (Malaysian National Institute of Health approval number NMRR-16-297-28898). F1 females were then crossed to males of the field strain. An ovitrap container lined with damp filter paper (diameter, 12 cm) was set as an oviposition site in each cage. After three days, the filter papers containing eggs were collected and allowed to air dry prior to use.

### 2.4. Backcrossing

For backcrossing, individual *w*AlbB infected females were sorted into glass tubes at the pupal stage. A total of 100 females emerged (aged 3–5 days) and were placed into a 24 × 24 × 24 cm sized cage. Wild males (aged 3–5 days) were put into the same cage at an equal ratio. A 10% sucrose solution with a vitamin-B complex was supplied as an energy source. The mosquitoes were left for mating in the cage for 2–3 days prior to blood feeding. An ovitrap container lined with damp filter paper (diameter, 12 cm) was set as an oviposition site in each cage. After three days, the filter papers containing eggs were collected and allowed to air dry prior to use.

### 2.5. Adult Bioassays

The adult mosquito bioassay was performed according to the standard WHO susceptibility or resistance test protocol [34,35]. A total of 15–20 female adults aged 3–5 days post-eclosion were used in the bioassays. Three test replicates and two controls were used. The adults were exposed to impregnated paper with diagnostic dosage, permethrin 0.25% or deltamethrin 0.03%. Insecticide impregnated papers were purchased from the Vector Control Research Unit, Universiti Sains Malaysia, Penang.

In the tests, *Ae*. *aegypti* or *Ae*. *albopictus* were introduced into each tube. Exposure tubes with permethrin or deltamethrin impregnated papers were stood vertically throughout the test. Knockdown was scored every minute for 5 min, followed by every 5 min for a 1 h exposure period. After exposure, the mosquitoes were transferred into paper cups and were supplied with cotton soaked in 10% sugar solution. Mortality was recorded after 24 h. Classification of resistance status was made based on pre-determined WHO guidelines in which mortality of <90% is characterized as resistant.

### 2.6. Assays for Sodium Channel Mutations Associated with Pyrethroid Resistance

#### 2.6.1. *Aedes aegypti*

Three sodium channel (*Vssc*) mutation sites were screened in male and female *Ae*. *aegypti* on five occasions from the laboratory colony used for release (Backcross 3 F3, F8, F9 and F13 and Backcross 5 F0) and on males and females from field samples collected in November 2016 and September 2019 from PKNS, Shah Alam (see above). Dead and surviving female mosquitoes from the WHO bioassays (B3F9, B5F0 and field samples for permethrin; B3F20, B5F3 and field samples for deltamethrin) were also screened. The codon positions relating to the mutation sites in this study are labelled as S989P, V1016G and F1534C according to the sequence of the most abundant splice variant of the house fly, *Musca domestica*, *Vssc* (GenBank accession nos. AAB47604 and AAB47605) [36]. These mutation sites are equivalent to those in other studies that are labelled as S996P, V1023G and F1565C based on the *Vssc* homologue in *Ae*. *aegypti*, the AaNav protein (GenBank accession no. EU399181) [13].

Molecular screening of each mutation site in *Ae*. *aegypti* was achieved using Custom TaqMan^®^ Single Nucleotide Polymorphism (SNP) Genotyping Assays (Life Technologies Corporation, Carlsbad, CA, USA) developed using sequence data from Wuliandari et al. [14]. Primers, probe sequences and amplicon lengths for each mutation site (989, 1016, 1534) are described by Endersby-Harshman et al. [22].

A LightCycler^®^ II 480 (Roche, Basel, Switzerland) real time PCR machine with a 384-well format was used to run three replicates of each SNP assay. The PCR Master Mix consisted of 40x TaqMan^®^ assay (0.174 µL), 2× KAPA Fast PCR Probe Force qPCR Master Mix (KAPABIOSYSTEMS, Cape Town, South Africa) (3.5 µL), ddH_2_O and genomic DNA diluted 1:5 in water (2 µL). Cycling conditions for the PCR were pre-incubation of 3 min at 98 °C (ramp rate 4.8 °C/s) followed by 40 cycles of amplification at 95 °C for 10 s (2.5 °C/s ramp rate) and 60 °C for 20 s (2.5 °C/s ramp rate) (Acquisition mode: single) with a final cooling step of 37 °C for 1 min (2.5 °C/s ramp rate). The Roche LightCycler^®^ 480 Software Version 1.5.1.62 was used to conduct endpoint genotyping.

#### 2.6.2. *Aedes albopictus*

The *Vssc* codon 1534 in S6, Domain III of *Ae*. *albopictus* was screened for mutations using a forward primer designed to bind in the exon which contains codon 1534: (Alb171F–5′CCGATTCGCGAGACCAACAT3′) and the reverse primer of Kasai et al. [37] (aegSCR8). An amount of 2 µL of genomic DNA, extracted using Chelex^®^ resin (Bio-Rad Laboratories, Hercules CA, USA) and diluted 1:10, was amplified in a 25 µL PCR mix that included final concentrations of ThermoPol buffer Mg-free (1x) (New England Biolabs, Ipswich, MA, USA), dNTPs (200 µM each) (Bioline, London, UK), MgCl_2_ (1.5 mM), 0.5 µM each of forward and reverse primers, 0.625 units of Immolase™ Taq polymerase (Bioline, London, UK), and PCR-grade H_2_O. Thermocycling conditions for the PCR were initial denaturation at 95 °C for 10 min, 35 cycles at 95 °C for 30 s, annealment at 52 °C for 45 s and extension at 72 °C for 45 s, followed by a final extension of 5 min at 72 °C before cooling to 10 °C. PCR amplicons (220 bp) were sent to 1st BASE DNA Sequencing (Selangor, Malaysia) for Sanger sequencing on a 3730xl DNA analyzer. Sequences (up to 180 bp) were aligned and analyzed using the program Geneious^®^ 11.1.4 (Biomatters, Ltd., Auckland, New Zealand).

### 2.7. Analyses

The odds ratio is a measure of association or relationship between two categorical variables. In this case, we use the odds ratio to assess whether there was an association between the categorical variables genotype and phenotype. The genotype for some of the tests is the *Vssc* mutation profile and the phenotype is whether the mosquito died or survived the diagnostic dose of permethrin or deltamethrin. For other tests, the phenotype is either male or female and, in some cases the odds ratio was determined as the odds of surviving as one genotype rather than another. Chi-squared tests of independence were conducted to explore relationships between categorical variables using IBM^®^ SPSS^®^ Statistics Version 24 and with probabilities determined by the Monte Carlo procedure.

## 3. Results

### 3.1. Adult Bioassays

**Figure 2:**
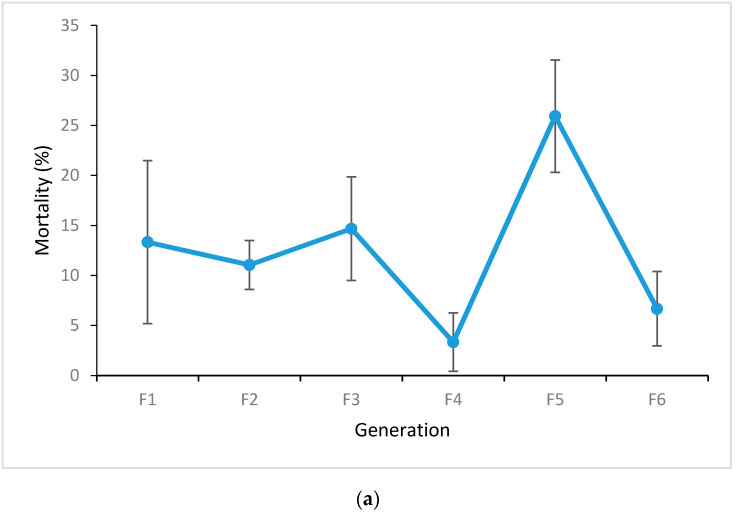

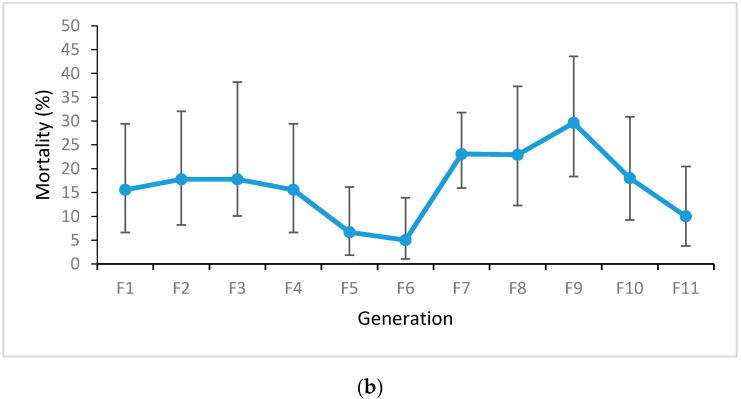
Mortality (%) of female *Ae*. *aegypti* adults from (**a**) the field strain from Shah Alam during the F1 to F6 generations in the laboratory, and (**b**) the *w*AlbB strain from B3F1 to B3F11 generations. Mortality was tested with WHO bioassay impregnated papers (permethrin 0.25%). Error bars represent 95% binomial confidence intervals.

Adult female *Ae*. *aegypti* from the field strain collected at Shah Alam without *Wolbachia* and tested at the F1–F3 generations were highly resistant to 0.25% permethrin delivered on impregnated filter papers in WHO diagnostic dose bioassays as reflected by a low mortality, although the mean mortality did tend to vary across generations (Figure 2a). The proportion surviving the treatment was higher than the proportion that died on all occasions, although there was some heterogeneity in numbers across multiple bioassays done on the F2 generation (contingency test, *χ*^2^ = 16.7, df = 3, *p* < 0.01). Over multiple generations in the laboratory (F1–F6), the Shah Alam field colony did vary in mortality (3.33 to 25.93%), but mortality did not change consistently over time. On the WHO bioassay scale, mortality of <90% is characterized as resistant. Permethrin bioassays of the *Wolbachia w*AlbB release strain of *Ae*. *aegypti* over multiple generations following backcrossing (B3F1–B3F11) also showed a high level of resistance, with mortality ranging from 5 to 29.63%, and again there was no consistent trend over time (Figure 2b).

Bioassays on the field strain of *Ae*. *albopictus* indicated resistance to permethrin based on the WHO categorization, but not to the same extent as *Ae*. *aegypti* (52% mortality (45.5–58.7, 95% confidence intervals) at 0.25% permethrin compared with 9.5% mortality of *Ae*. *aegypti*). However, no significant difference between proportions of these species that were dead or survived was detected (*χ*^2^ = 2.09, df = 1, *p* > 0.05).

Resistance to deltamethrin (0.03%) was detected by bioassays of *Ae*. *aegypti*. The laboratory colony at B3F20 showed 84.8% mortality, which dropped somewhat to 63.81% after further backcrossing (B5F3). A recent field colony, established from mosquitoes collected from Shah Alam where releases were terminated about 1.5 years ago and then reared for four generations in the lab (i.e., at the F4 generation), showed a similar mortality of 71.8%.

### 3.2. Assays for Sodium Channel Mutations Associated with Pyrethroid Resistance

Genotypes were compared among surviving and dead female *Ae*. *aegypti* from Shah Alam after bioassays with permethrin. These mosquitoes were tested prior to release of *Wolbachia*-infected mosquitoes, and showed the presence of three *Vssc* genotypes (GG/TT/CC, TT/GG/TT, TG/TG/TC—order 1016/1534/989) (Table 1), each of which would be expected to confer some level of resistance to pyrethroids. The odds of having the mutant genotype (GG or CC) and surviving were not different from survival without these mutations when considering single mutation sites at a time (1016 or 989: odds of surviving if mutant homozygote = 0.12 (0.01–1.03, 95% c.i.) and 1534 OR = 0.89 (0.35–2.29, 95% c.i.), NS = not statistically significant at α = 0.05–c.i. values encompass 1.0). However, if genotypes for the three mutation sites are considered together, the odds of surviving 0.25% permethrin were significantly higher for a triple heterozygote than for a wildtype genotype (Table 2). TT/GG/TT individuals also had greater odds of survival than wildtype mosquitoes (α = 0.05–c.i. values do not encompass 1.0) (Table 2).

The odds of surviving 0.03% deltamethrin were greatly enhanced for mosquitoes with the mutant genotypes TT/GG/TT or TG/TG/TC compared with wildtype or genotypes TG/TT/TC or TT/TG/TT (Table 3b).

**Figure 3:**
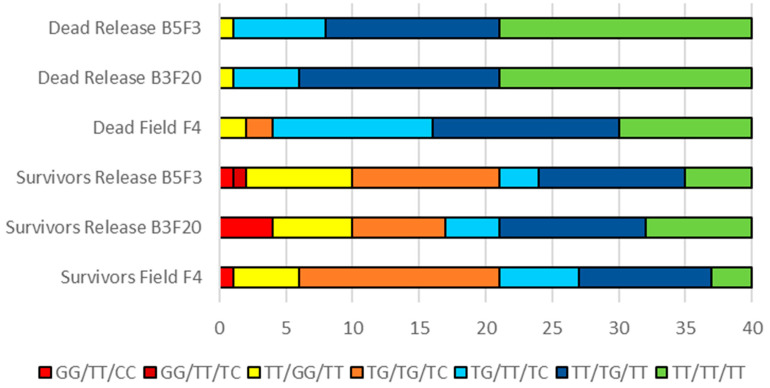
Proportion of each *Vssc* genotype in 40 samples of dead and surviving *Aedes aegypti* females from bioassays with deltamethrin (type II pyrethroid) (0.03%). Mutations listed in order 1016/1534/989 (in all cases T = wildtype).

Odds ratios could not be calculated for mosquitoes with genotype GG/TT/CC that were found in relatively low numbers in the sample, because no dead mosquitoes had this genotype (Figure 3).

**Figure 4:**
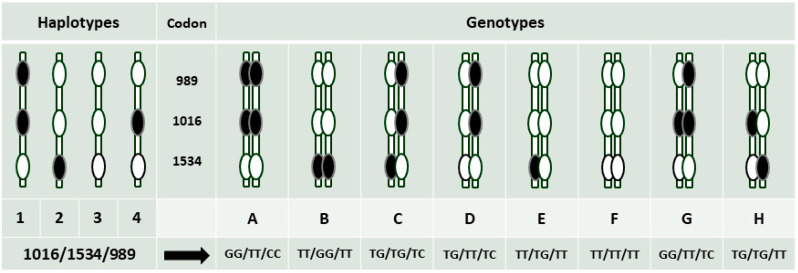
Putative *Vssc* haplotypes and observed genotypes in field (Shah Alam) (A–F) and IMR colony samples (A–G) of *Aedes aegypti*.

In a comparison of *Vssc* genotypes found in the field (Shah Alam 2016) and colony (B3F3 IMR) samples of *Ae*. *aegypti* (Table 4), four *Vssc* genotypes were observed in both the field and colony samples (A = GG/TT/CC, B = TT/GG/TT, C = TG/TG/TC, E = TT/TG/TT) (order of mutation sites 1016/1534/989). An additional genotype (D = TG/TT/TC) was found in mosquitoes from the colony. These five genotypes can be formed from three putative haplotypes (Figure 4). The frequency of the three most common genotypes in the field mosquitoes (A, B, C) is similar to that in the release colony (*χ*^2^ = 0.124, df = 2, *p* = 0.940).

In both the Shah Alam field sample and the colony (B3F3), there were more female than male heterozygotes (TG/TG/TC) (Table 4). 

Overall differences in genotype distribution between the sexes in the field were only marginally significant (*χ*^2^ = 6.014, df = 2, *p* = 0.049) for the common genotypes A, B and C. In the B3F3 colony, however, the genotype distribution (Figure 4A–C,) differed significantly between the sexes (*χ*^2^ = 14.882, df = 2, *p* = 0.001). The odds of having genotype TT/GG/TT and being male were significantly different from expectations (*p* < 0.05) in both the field and the colony, but the odds ratio in the colony was higher (odds of having mutations GG at 1534 and being male: field (Shah Alam), OR = 3.27 (1.21–8.84, 95% c.i.); colony B3F3, OR = 15.55 (3.29–73.42, 95% c.i.)). These odds ratios compared proportions of male TT/GG/TT out of males of other genotypes with the proportion of female TT/GG/TT out of females of other genotypes.

A test for the Hardy–Weinberg equilibrium (HWE) of the genotypes at the three *Vssc* mutation sites in *Aedes aegypti* from the field (Shah Alam), the laboratory (B3F3 IMR) and the bioassayed individuals (dead and alive) showed that the genotypes in the bioassay samples were not in HWE but the other samples were in HWE (Table S1). This deviation may reflect the fact that equal numbers of dead and surviving mosquitoes were genotyped, which meant that mosquito sampling in the bioassays was biased.

Comparison of *Vssc* mutations over time in the IMR *Wolbachia* release colony of *Ae*. *aegypti* showed changes in genotype distribution over generations in the B3 backcross (Table 5), which were significant by a contingency test for both females (*χ*^2^ = 36.20, df = 10, *p* < 0.001) and males (*χ*^2^ = 27.08, df = 10, *p* = 0.002).

**Figure 5:**
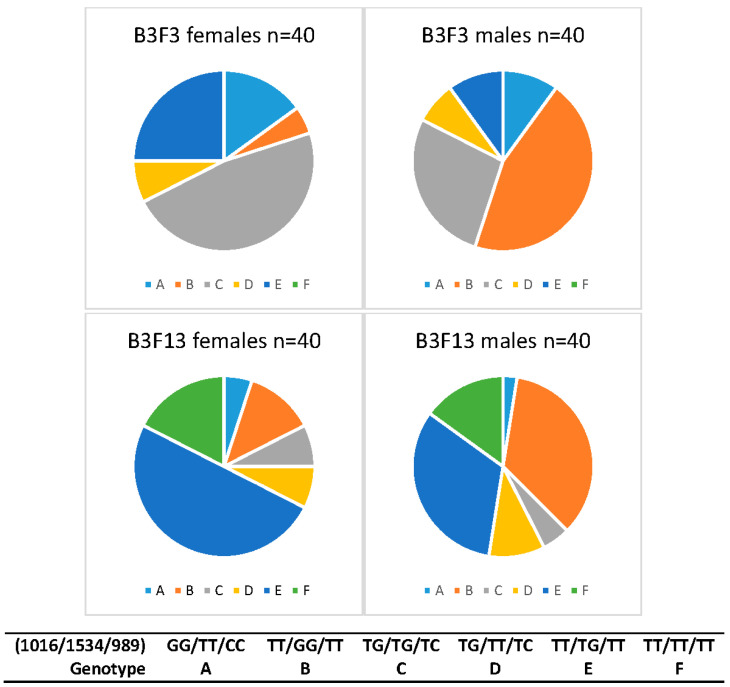
Changes in frequency of *Vssc* genotypes between B3F3 and B3 F13 (ten generations) of the IMR laboratory colony (*w*AlbB infected) and differences between female and male *Aedes aegypti*.

Wildtype individuals (TT/TT/TT) were not found in the B3F3 baseline population, but were common in both sexes in B3F8. TT/TG/TT was the most common female genotype in B3F8, whereas the triple heterozygote (TG/TG/TC) had been the most common in B3F3. TT/GG/TT remained the most common genotype in males between B3F3 and B3F8, but the prevalence of triple heterozygotes declined and TT/TG/TT increased as it did in females. After ten generations without backcrossing to field mosquitoes (B3F3 to B3F13), changes in the *Vssc* mutation profile became apparent (Figure 5). No wildtype mosquitoes were observed in the baseline population, but at B3F13, they comprised 16% of the samples screened (n = 80). The triple heterozygote decreased in frequency (38% to 6%) and the 1016/989 mutation (genotype A), which did not occur in large numbers, also decreased by two thirds. The 1534 mutation (genotype B) was maintained in the laboratory colony, but the heterozygote (genotype E) increased (18% to 41%). These results point to a decrease in the resistant genotypes and alleles.

A fourth and fifth backcross was undertaken, and in the B5F0 mosquitoes, the homozygous 1016 mutation was not found in the sample of males (Table 5). After further backcrossing, the sex difference in the frequency of the 1534 mutation was no longer evident, and the prevalence of the TT/TG/TT genotype decreased overall. Wildtype individuals (TT/TT/TT) were not observed in the backcrossed sample, while the triple heterozygote, TG/TG/TC, became more abundant. The backcrossing introduced two new genotypes to the laboratory colony, H = TG/TG/TT (Table 1) and G = GG/TT/TC (Table 3a), both of which include a fourth haplotype (H4—Figure 4) not identified in the field sampling, but clearly present in the wild males used for the cross. The additional backcross generations therefore reintroduced resistant alleles into the population.

A comparison of the distribution of *Vssc* genotypes (A, B, C) at the field site, Shah Alam, in 2016 before the *Wolbachia* release and in 2019 after release, showed that the distribution of these genotypes had remained the same (*χ*^2^ = 3.91, df = 2, *p* = 0.141) (Table 6).

### 3.3. Aedes albopictus

The DNA sequences for 27 individuals of *Aedes albopictus* from AU2, Keramat, for a small section of the voltage-sensitive sodium channel gene (*Vssc*) from S6, domain III, were obtained (Supplementary Material S1). The wildtype state at codon 1534 was TTC (phenylalanine). A total of 23 individuals showed this genotype (GenBank Accession numbers: (MK992016, MK992018, MK992020-MK992029, MK992031-MK992033, MK992035-MK992042). Sample E09 (MK992030) showed a non-synonymous homozygous mutation at codon 1534 (TTG/TTG) that would code for leucine. Samples A10 (MK992017), B07 (MK992019), and F10 (MK992034) were heterozygotes at codon 1534 (TTC/TTG) (IUPAC code S for C/G appears in the sequence).

Synonymous mutations (TTT and TTC) were found at codon 1528, some of which comprised heterozygotes (IUPAC code Y for C/T) (13 individuals). Sample E07 (MK992029) was heterozygous at position three in codon 1539 (IUPAC code Y for C/T), but it is a synonymous mutation. Samples A01 (MK992016), B05 (MK992018), B10 (MK992021), C6 (MK992024) and E4 (MK992028) were heterozygotes at codon 1544 position three (IUPAC code Y for C/T). The T is a synonymous mutation (isoleucine). The same five samples were also heterozygous at codon 1561, position 3 (IUPAC code K for G/T), another synonymous mutation (serine). Another heterozygote occurred at position three, codon 1547, in sample B08 (MK992020) (IUPAC code Y for C/T). The alternatives are again synonymous (isoleucine). Sample G09 (MK992038) was heterozygous at codon 1582, position three (IUPAC code R for A/G), but the mutation is synonymous (leucine).

## 4. Discussion

Pressure from pyrethroid insecticide applications can be intensive in regions where mosquitoes involved in transmission of dengue (*Ae*. *aegypti* and *Ae*. *albopictus*) are common, and selection under these conditions seems inevitable, with very few exceptions where pesticide applications have been restricted [38]. Alternative dengue control programs, which involve release of dengue-blocking *Wolbachia*-infected mosquitoes to replace natural populations, can reduce insecticide applications as mosquitoes reduce their virus-transmitting competence [26,39]. However, during the establishment phase of these programs, it is likely that conventional control options such as pesticide application will continue to be required, potentially jeopardizing survival of the laboratory-reared mosquitoes and threatening the success of *Wolbachia* release programs [31]. To mitigate this problem, *Wolbachia* mosquitoes are backcrossed to field individuals multiple times to ensure they have an equivalent genetic background [27] which, amongst other traits, will include resistance to the level of insecticide pressure experienced at the release sites. However, resistance can be lost from release populations, even after backcrossing, if there are costs associated with resistance alleles [30,31].

A *Wolbachia* mosquito release program for *Ae*. *aegypti* conducted around Kuala Lumpur in Malaysia from 2016 to the present day has been successful both in establishment of the *Wolbachia* mosquitoes and in reducing the number of dengue cases in release areas [25]. Throughout the establishment of the program, there was an opportunity to study various aspects of mosquito releases. Our study focused on the effects of continued pyrethroid applications around the release areas, the resistance genotypes and phenotypes of field and laboratory mosquitoes and benefits of regular backcrossing to field mosquitoes compared with long-term mosquito rearing as a closed population.

### 4.1. Resistance in the Dengue Vector Mosquitoes in Kuala Lumpur

We found phenotypic resistance to both type I and II pyrethroids in *Ae*. *aegypti* and resistance to at least type I pyrethroids in *Ae*. *albopictus* in the field. Resistance to both type I and II pyrethroids was also observed in release colony samples of *Ae*. *aegypti*. Using three *Vssc* mutations as a proxy for pyrethroid resistance in general was less successful with the type I pyrethroid, permethrin, than with type II (deltamethrin), as there was only a weak correspondence between the presence of some genotypes and survival after permethrin exposure. The triple heterozygote (V1016G/F1534C/S989P) had a small survival advantage over the wildtype genotype (V1016/F1534/S989) and TT/GG/TT individuals also had a somewhat greater odds of survival than wildtype mosquitoes. In contrast, the odds of surviving deltamethrin (0.03%) exposure were greatly enhanced by presence of the *Vssc* mutations, particularly the triple heterozygote and the homozygous mutations (GG/TT/CC and TT/GG/TT) which is consistent with findings of electrophysiological studies of these mutations expressed in *Xenopus* oocytes [13].

In bioassays, *Aedes aegypti* showed a higher level of resistance to permethrin compared with *Ae*. *albopictus*. This result is consistent with other studies [40] and most likely reflects a lower level of exposure to pyrethroids experienced by *Ae*. *albopictus,* as its habitat extends beyond the dwellings of humans where most insecticide applications take place [4]. With increased urbanization and increased exposure to pyrethroids by *Ae*. *albopictus*, the lower resistance levels in this species are likely to change and, in fact, appear to be doing so within southern and eastern China at present [41,42].

A newly discovered mutation, F1534L, in *Ae*. *albopictus* from Kuala Lumpur may be an indication that selection for higher levels of target-site resistance is underway in Malaysia as well. The F1534L mutation found in the *Ae*. *albopictus* samples in this study is the first sodium channel mutation to be reported from this species in Malaysia, even though extensive surveying was conducted in 2010 [4]. It appears that the resistance mutation in *Ae*. *albopictus* has increased to a detectable frequency since the 2010 samples were taken by Ishak et al. [4]. Ishak et al. [12] demonstrated that metabolic resistance caused by upregulation of the cytochrome P450, CYP6P12, was the main cause of resistance in *kdr*-free *Ae*. *albopictus*, but six years later, monitoring for *kdr* also may be justified given that we have now detected a *Vssc* mutation.

A F1534L mutation has been found in *Ae*. *albopictus* in Florida, USA (2011) [43], but involves a TTC to CTC transition in the codon rather than the TTC to TTG mutation that we have detected. An F1534L mutation (TTC to TTG) has also been detected in *Ae*. *albopictus* from Guangzhou, China (2014), Shenzhen, China (2014) and from Arco, Italy (2011) [44]. The F1534L mutation is also now present in *Ae*. *albopictus* in Beijing [42]. Yan et al. [45] have made a functional study of this mutation and found that it reduced the *Vssc* sensitivity to type I, but not type II pyrethroids. Even so, Li et al. [46] found a significant association between F1534L and resistance to deltamethrin in *Ae*. *albopictus*.

*Vssc* mutations were found more commonly at codon 1534 than at the 1016 and 989 codons in *Ae*. *aegypti* from the release area around Kuala Lumpur. This result is consistent with that of Ishak et al. [4] who found the same trend in mosquitoes across Malaysia. The results are very different from similar studies of *Ae*. *aegypti* from Kedah and Johor states (the northern and southern regions of Malaysia) [47] as well as Yogyakarta [14], in which mutations at 1016 and 989 were common, but 1534 was rare. Within southeast Asia and the Indo-Pacific, the F1534C mutation in *Ae*. *aegypti* predominates also in Taiwan, Vietnam, Sri Lanka and Fiji, whereas in Bali (Indonesia) and Vanuatu, V1016G with S989P are the only mutations found [22].

### 4.2. Comparison between Vssc Genotypes in the Release Colony and the Field

In general terms, the *Wolbachia* release colony and field *Ae*. *aegypti* samples are very similar with respect to frequency of V1016G and S989P mutations, but, as mentioned above, these mutations occur at lower frequencies than F1534C in the tested samples. The *Wolbachia* release colony and field mosquitoes are also similar with respect to overall F1534C mutation frequency, although fewer homozygotes and more heterozygotes are found in the colony. This could have some impact on resistance level. The effects of *Vssc* mutations on fitness parameters not related to insecticide resistance are not known for *Ae*. *aegypti*, but a *Vssc* mutation in *Anopheles gambiae*, L1014F, has been shown to confer a male mating advantage in heterozygous individuals compared with males homozygous for the mutation and wildtype males [48].

The *Wolbachia* release colony at B3F3 had one combined genotype not found in the field (TG/TT/TC genotype D, which looks like it received a wildtype haplotype (H3) from one parent and the H1 haplotype from the other). We found no individuals in the colony or field samples which were wildtype at all three loci at generation B3F3, however, with haplotype H3 being present in the colony, it is likely that they may exist at low frequency. Genotype E, found in the field, must also have had one parent that was wildtype or had a wildtype haplotype. In later samples (Field 2019), we found homozygous wildtype individuals in the field, meaning that there will still be some susceptibility to pyrethroid insecticides in *Ae*. *aegypti* in Kuala Lumpur. Wildtype homozygotes were also found in the release colony from B3F8 onwards, suggesting that this closed colony was changing over time with a low-frequency genotype reaching detectable levels.

### 4.3. Changes in Genotype Frequencies in the Release Strain over Time: Implications for Field Survival

We detected a tendency for male *Ae*. *aegypti* in the field, but particularly the laboratory colony, to have a higher frequency of the F1534C homozygous mutation than females. Such a difference cannot be attributed to sex-linkage, as the *Ae*. *aegypti* sex-determining region is located on chromosome 1 and the *Vssc* gene is on chromosome 3 [36]. If males and females were coming from different populations as happens at each backcross event, this might provide an explanation. Otherwise, there might be environmental conditions where males and females are selected differently; perhaps a lethal effect of this mutation in females gives a sex-specific fitness cost. The conditions of colony rearing could make this cost higher than in the field. Though an interesting phenomenon, it only appears to occur sporadically, making further investigations difficult.

Between B3F3 and B3F8 of the *Ae*. *aegypti Wolbachia* release colony, there was a tendency for more susceptible genotypes to increase in frequency, and this continued further at B3F13 and B3F20. Resistance to type I pyrethroids is likely to have been maintained in the colony to some extent by 1534 in males, while the 1016 component was being lost. Though the colony was still classified as resistant according to WHO, mortality caused by 0.03% deltamethrin was high at 84.8% in the closed *Ae*. *aegypti* colony at B3F20 compared with mortality levels with 0.25% permethrin. Resistance to this type II pyrethroid was clearly regained after backcrossing, as mortality dropped to 63.8% (B5F3) and became equivalent to that of the recent field collection (F4), which showed mortality of 71.8% to deltamethrin in the same bioassay. In a newly backcrossed population (B5F0), the 1016 mutation did not increase in frequency and was not found at all in the sample of males. Backcrossing removed the sex difference in frequency of the 1534 mutation. The prevalence of the TT/TG/TT genotype decreased overall and wildtype individuals TT/TT/TT were not observed in the backcrossed sample despite having occurred at a high frequency in B3F3. By B5F3, however, wildtype homozygotes were detectable again. The implications of long-term rearing without backcrossing on resistance status of a release colony are demonstrated. We note the previous evidence for loss of resistance in *w*Mel *Wolbachia* infected lines in Brazil, which was associated with a failure to establish this infection in the field [30,31].

Releases of *Wolbachia* mosquitoes in the study area occurred between May 2017 and November 2017 as weekly events for a block of 26 weeks followed by a four-month gap and a second block of weekly releases for 31 weeks [25] using early generations of backcrossed material. Two years after release, *Wolbachia* infection of the mosquito population at the sites remained at >90% [25] and *Vssc* mutations had not changed in distribution from our original screening before releases in 2016, demonstrating the value of keeping equivalent resistance mechanisms in the release population by backcrossing to mosquitoes from the field. While we focused on *Vssc* mutations and target-site resistance as a proxy measurement for pyrethroid resistance overall, metabolic resistance mechanisms in *Ae*. *aegypti* populations in Malaysia also play a significant role [4,11,12]; thus, analysis of these mechanisms would be a useful addition to further understand backcrossing requirements for *Wolbachia* release programs in both *Ae*. *aegypti* and *Ae*. *albopictus*. In such analyses, it would be useful to make comparisons and undertake crosses to the well-characterized susceptible “Rockefeller” strain, which would also allow for more direct comparisons with other bioassay studies where this strain is often characterized.

## 5. Conclusions

We have identified *Vssc* mutations associated with pyrethroid resistance in populations of *Aedes aegypti* from Kuala Lumpur, Malaysia. Mosquitoes in two genotype classes demonstrated a type I pyrethroid resistance advantage over wildtype mosquitoes when exposed to 0.25% permethrin. This resistance advantage was even more pronounced with a type II pyrethroid, deltamethrin (0.03%). A *Vssc* mutation in *Aedes albopictus* (F1534L), found for the first time in mosquitoes from Malaysia, suggests that selection for pyrethroid resistance in this second dengue vector species may be in progress.

Three *Vssc* haplotypes predominate in *Ae*. *aegypti* in this region and all persist in colonies with regular backcrossing to mosquitoes from the field. Changes in *Vssc* genotype frequency in closed populations of *Ae*. *aegypti* maintained for multiple generations in laboratory culture suggest different fitness costs associated with the genotypes, some of which may be associated with the sex of the mosquito. Following population replacement of *Ae*. *aegypti* by *Wolbachia* mosquitoes for dengue control using backcrossed populations, we found that the *Vssc* mutations have persisted at pre-release levels. The results point to the importance of *Vssc* mutations in pyrethroid resistance in mosquito populations and the need for regular backcrossing with male mosquitoes from the field to maintain similarity of genetic background and population integrity during *Wolbachia* mosquito releases.

## Figures and Tables

**Table 1:** *Vssc* genotypes of survivors and dead female *Aedes aegypti* from the field and the laboratory release colony from WHO bioassays with permethrin (0.25%) (order of mutation sites 1016/1534/989) (Field = Shah Alam 2016 pre-release and Shah Alam 2019 after release; the laboratory release colony was tested at B3F9 and B5F0).

		Genotype Frequency (%) (n)						
		GG/TT/CC	TT/GG/TT	TG/TG/TC	TG/TT/TC	TT/TG/TT	TT/TT/TT	TG/TG/TT
	Gen	A	B	C	D	E	F	H
Survivors	Field 2016	2.5 (1)	30.0 (12)	67.5 (27)	0.0 (0)	0.0 (0)	0.0 (0)	0.0 (0)
Dead	Field 2016	17.5 (7)	32.5 (13)	50.0 (20)	0.0 (0)	0.0 (0)	0.0 (0)	0.0 (0)
Survivors	Field 2019	15.0 (6)	35.0 (14)	45.0 (18)	2.5 (1)	2.5 (1)	0.0 (0)	0.0 (0)
Dead	Field 2019	12.5 (5)	37.5 (15)	35.0 (14)	0.0 (0)	12.5 (5)	2.5 (1)	0.0 (0)
Survivors	Release B3F9	2.5 (1)	15.0 (6)	15.0 (6)	5.0 (2)	52.5 (21)	10.0 (4)	0.0 (0)
Dead	Release B3F9	2.5 (1)	10.0 (4)	10.0 (4)	10.0 (4)	35.0 (14)	32.5 (13)	0.0 (0)
Survivors	Release B5F0	2.5 (1)	42.5 (17)	27.5 (11)	5.0 (2)	22.5 (9)	0.0 (0)	0.0 (0)
Dead	Release B5F0	5.0 (2)	35.0 (14)	20.0 (8)	0.0 (0)	35.0 (14)	0.0 (0)	5.0 (2)

**Table 2:** Odds ratio comparisons of genotypes of *Aedes aegypti* (n = 322) from field and laboratory colonies (permethrin bioassay results combined). Odds ratio (OR) of surviving 0.25% permethrin with a specific genotype (order of mutation sites 1016/1534/989) (n = number of each genotype) are presented.

Odds of Surviving		n	OR	95% Confidence Interval		OR Significant α = 0.05
If:	Rather Than:			Lower	Upper	
TG/TG/TC	TT/TT/TT	108, 18	4.72	1.46	15.27	*
TG/TT/TC	TT/TT/TT	9, 18	4.38	0.78	24.47	
TT/GG/TT	TT/TT/TT	95, 18	3.73	1.14	12.16	*
TT/TG/TT	TT/TT/TT	64, 18	3.29	0.98	11.08	
GG/TT/CC	TT/TT/TT	24, 18	2.10	0.53	8.39	
TG/TG/TC	GG/TT/CC	108, 24	2.25	0.90	5.58	
TG/TT/TC	GG/TT/CC	9, 24	2.08	0.44	9.84	
TT/GG/TT	GG/TT/CC	95, 24	1.78	0.71	4.45	
TT/TG/TT	GG/TT/CC	64, 24	1.57	0.60	12.09	
TG/TG/TC	TT/GG/TT	108, 95	1.27	0.73	2.20	
TT/GG/TT	TT/TG/TT	95, 64	1.13	0.60	2.14	
TG/TG/TC	TG/TT/TC	108, 9	1.08	0.27	4.24	

* Significant (α = 0.05).

**Table 3:** (**a**) *Vssc* genotypes of survivors and dead female *Aedes aegypti* from the field and the laboratory release colony from WHO bioassays with deltamethrin (0.03%) (order of mutation sites 1016/1534/989) (Field = Shah Alam F4, laboratory colony was tested at B3F20 and B5F3). (**b**) Odds ratios (OR) of surviving deltamethrin (type II pyrethroid) when a particular genotype is compared with another are also given.

**(a)**		**Genotype Frequency (%) (n)**						
		**GG/TT/CC**	**TT/GG/TT**	**TG/TG/TC**	**TG/TT/TC**	**TT/TG/TT**	**TT/TT/TT**	**GG/TT/TC**
	**Generation**	**A**	**B**	**C**	**D**	**E**	**F**	**G**
Survivors	Field F4	2.5 (1)	12.5 (5)	37.5 (15)	15.0 (6)	25.0 (10)	7.5 (3)	0.0 (0)
Dead		0.0 (0)	5.0 (2)	5.0 (2)	30.0 (12)	35.0 (14)	25.0 (10)	0.0 (0)
Survivors	Release B3F20	10.0 (4)	15.0 (6)	17.5 (7)	10.0 (4)	27.5 (11)	20.0 (8)	0.0 (0)
Dead		0.0 (0)	2.5 (1)	0.0 (0)	12.5 (5)	37.5 (15)	47.5 (19)	0.0 (0)
Survivors	Release B5F3	2.5 (1)	20.0 (8)	27.5 (11)	7.5 (3)	27.5 (11)	12.5 (5)	2.5 (1)
Dead		0.0 (0)	2.5 (1)	0.0 (0)	17.5 (7)	32.5 (13)	47.5 (19)	0.0 (0)
**(b)**	**Odds of Surviving**		**n**	**OR**	**95% Confidence Interval**		**OR Significant** **α = 0.05**	
	**if:**	**rather than:**			**Lower**	**Upper**		
	TT/GG/TT	TG/TG/TC	23, 35	0.29	0.05	1.72		
	TT/GG/TT	TG/TT/TC	23, 37	8.77	2.46	31.29	*	
	TT/GG/TT	TT/TG/TT	23, 74	6.23	1.93	20.13	*	
	TT/GG/TT	TT/TT/TT	23, 64	14.25	4.22	48.16	*	
	TG/TG/TC	TG/TT/TC	35, 37	30.46	6.28	147.72	*	
	TG/TG/TC	TT/TG/TT	35, 74	21.66	4.83	97.02	*	
	TG/TG/TC	TT/TT/TT	35, 64	49.50	10.66	229.83	*	
	TG/TT/TC	TT/TG/TT	37, 74	0.71	0.31	1.61		
	TG/TT/TC	TT/TT/TT	37, 64	1.63	0.67	3.92		
	TT/TG//TT	TT/TT/TT	74, 64	2.29	1.10	4.74	*	

* Significant (α = 0.05).

**Table 4:** Comparison of *Vssc* genotypes found in field (Shah Alam, 2016) and colony (B3F3 IMR) samples of *Aedes aegypti* (order of mutation sites 1016/1534/989) at the time of initial *Wolbachia* releases.

Genotype Frequency (%) (n)						
	GG/TT/CC	TT/GG/TT	TG/TG/TC	TG/TT/TC	TT/TG/TT	n
	A *	B *	C *	D	E	
Field Female	15.00 (6)	20.00 (8)	60.00 (24)	0.0 (0)	5.00 (2)	40
Field Male	12.50 (5)	45.00 (18)	37.50 (15)	0.0 (0)	5.00 (2)	40
Total	13.75 (11)	32.50 (26)	48.75 (39)	0.0 (0)	5.00 (4)	80
Colony Female	15.00 (6)	5.00 (2)	47.50 (19)	7.50 (3)	25.00 (10)	40
Colony Male	10.00 (4)	45.00 (18)	27.50 (11)	7.50 (3)	10.00 (4)	40
Total	12.50 (10)	25.00 (20)	37.50 (30)	7.50 (6)	17.50 (14)	80

* (Total field/total colony: *χ*^2^ = 0.124, df = 2, *p* = 0.940; field female/field male: *χ*^2^ = 6.014, df = 2, *p* = 0.049; colony female/colony male: *χ*^2^ = 14.882, df = 2, *p* = 0.0006).

**Table 5:** Comparison of *Vssc* mutations across four generations of the IMR colony of *Aedes aegypti* (B3F3, B3F8, B3F13, B5F0) (order of mutation sites: 1016/1534/989).

	Genotype Frequency (%) (n)						
Genotype	GG/TT/CC	TT/GG/TT	TG/TG/TC	TG/TT/TC	TT/TG/TT	TT/TT/TT	n
	A	B	C	D	E	F	
B3F3 Female	15.0 (6)	5.0 (2)	47.5 (19)	7.5 (3)	25.0 (10)	0.0 (0)	40
B3F3 Male	10.0 (4)	45.0 (18)	27.5 (11)	7.5 (3)	10.0 (4)	0.0 (0)	40
B3F8 Female	0 (0)	13.3 (4)	20.0 (6)	13.3 (4)	43.3 (13)	10.0 (3)	30
B3F8 Male	7.1 (2)	46.4 (13)	3.6 (1)	7.1 (2)	21.4 (6)	14.3 (4)	28
B3F13 Female	5.0 (2)	12.5 (5)	7.5 (3)	7.5 (3)	50.0 (20)	17.5 (7)	40
B3F13 Male	2.5 (1)	35.0 (14)	5.0 (2)	10.0 (4)	32.5 (13)	15.0 (6)	40
B5F0 Female	7.5 (3)	25.0 (10)	37.5 (15)	7.5 (3)	22.5 (9)	0.0 (0)	40
B5F0 Male	0.0 (0)	27.5 (11)	37.5 (15)	22.5 (9)	12.5 (5)	0.0 (0)	40

**Table 6:** Comparison of *Vssc* genotypes found in the field (PKNS Shah Alam) in 2016 (pre-release) and 2019 (post-release of *Wolbachia*) in *Ae*. *aegypti.*

		Genotype Frequency (%) (n)						
		GG/TT/CC	TT/GG/TT	TG/TG/TC	TG/TT/TC	TT/TG/TT	TT/TT/TT	n
		A	B	C	D	E	F	
Field Female	2016	15.00 (6)	20.00 (8)	60.00 (24)	0.0 (0)	5.00 (2)	0.00 (0)	40
Field Male		12.50 (5)	45.00 (18)	37.50 (15)	0.0 (0)	5.00 (2)	0.00 (0)	40
Total		13.75 (11)	32.50 (26)	48.75 (39)	0.0 (0)	5.00 (4)	0.00 (0)	80
Field Female	2019	15.00 (6)	32.50 (13)	35.00 (14)	2.50 (1)	12.50 (5)	2.50 (1)	40
Field Male		25.00 (10)	40.00 (16)	27.50 (11)	0.00 (0)	7.50 (3)	0.00 (0)	40
Total		20.00 (16)	36.25 (29)	31.25 (25)	1.25 (1)	10.00 (8)	1.25 (1)	80

Note: Distribution of genotypes A, B, C in 2016 compared with 2019; *χ*^2^ = 3.91, df = 2, *p* = 0.141.

## Supplementary Materials

The following are available online at https://www.mdpi.com/2075-4450/11/8/529/s1, Table S1: Test for Hardy–Weinberg equilibrium of genotypes at three Vssc mutation sites in Aedes aegypti from the field (Shah Alam), the laboratory (B3F3 IMR) and bioassayed female individuals (dead and alive); Supplementary Material S1: DNA sequences for 27 individuals of Aedes albopictus for a small section of the voltage-sensitive sodium channel gene (Vssc) from S6, domain III from Shah Alam.
