# Supplementary material for: Characterization of Sodium Channel Mutations in the Dengue Vector Mosquitoes Aedes aegypti and Aedes albopictus within the Context of Ongoing Wolbachia Releases in Kuala Lumpur, Malaysia
Source: Insects. 2020 Aug 13;11(8):529. doi: 10.3390/insects11080529 (PMC7469223; doi:10.3390/insects11080529)
Supplement: Supplementary file 1 [file insects-11-00529-s001.pdf]

Supplementary Table S1. Tests for Hardy-Weinberg equilibrium of genotypes at three *Vssc* mutation sites in *Aedes aegypti* from the field (Shah Alam), the laboratory (B3F3 IMR) and bioassayed female individuals (dead and alive).

**Mutation/Population**		**Genotype**		**n**	**X^2**	**P-value**
**V1016G**	**TT**	**TG**	**GG**		**df=1**	**(0.05)**
**Field**	30	39	11	80	0.089	0.766
**Colony**	34	36	10	80	0.010	0.922
**Bioassay**	25	47	8	80	4.253	**0.039**
**F1534C**	**TT**	**TG**	**GG**			
**Field**	11	43	26	80	1.043	0.307
**Colony**	16	44	20	80	0.845	0.358
**Bioassay**	8	47	25	80	4.253	**0.039**
**S989P**	**TT**	**TC**	**CC**			
**Field**	30	39	11	80	0.089	0.766
**Colony**	34	36	10	80	0.010	0.922
**Bioassay**	25	47	8	80	4.253	**0.039**

Supplementary Material S1

DNA sequences for 27 individuals of *Aedes albopictus* for a small section of the voltage-sensitive sodium channel gene (*Vssc*)from S6, domain III from Shah Alam, Selangor, Malaysia. The wildtype state at codon 1534 is TTC (Phenylalanine)and is shaded in yellow. Sample E09 has a non-synonymous homozygous mutation at codon 1534 and would code for Leucine (shaded in green). Samples A10, B07, F10 are heterozygotes at codon 1534 (TTC/TTG) and are shaded in blue (IUPAC code S for C/G appears in the sequence). Synonymous mutations (TTT and TTC) were found at codon 1528, some of which comprised heterozygotes (IUPAC code Y for C/T) and are highlighted in grey. Sample E07 was heterozygous at position three in codon 1539 (IUPAC code Y for C/T) and is highlighted in grey, but it is a synonymous mutation. Samples A01, B05, B10, C6 and E4 were heterozygotes at codon 1544 position three (IUPAC code Y for C/T) and are highlighted in grey. The T is a synonymous mutation (Isoleucine). The same five samples were also heterozygous at codon 1561, position 3 (IUPAC code K for G/T), another synonymous mutation (Serine)and shaded in red. Another heterozygote occurs at position three, codon 1547 in sample B08 (IUPAC code Y for C/T). The alternatives are again synonymous (Isoleucine). Sample G09 is heterozygous at codon 1582, position three (IUPAC code R for A/G), but the mutation is synonymous (Leucine). Bases unable to be called accurately (N) are highlighted in pink.

```
                  1        10        20        30        40        50        60
IMRSASA01     CTACATGTACCTCTACTTYGTGTTCTTCATCATCTTCGGGTCGTTCTTCACCCTCAACCT
IMRSASA10     CTACATGTACCTCTACTTTGTGTTCTTCATCATCTTSGGGTCGTTCTTCACCCTCAACCT
IMRSASB05     CTACATGTACCTCTACTTYGTGTTCTTCATCATCTTCGGGTCGTTCTTCACCCTCAACCT
IMRSASB07     CTACATGTACCTCTACTTTGTGTTCTTCATCATCTTSGGGTCGTTCTTCACCCTCAACCT
IMRSASB08     CTACATGTACCTCTACTTYGTGTTCTTCATCATCTTCGGGTCGTTCTTCACCCTCAACCT
IMRSASB10     CTACATGTACCTCTACTTYGTGTTCTTCATCATCTTCGGGTCGTTCTTCACCCTCAACCT
IMRSASC01     CTACATGTACCTCTACTTYGTGTTCTTCATCATCTTCGGGTCGTTCTTCACCCTCAACCT
IMRSASC03     CTACATGTACCTCTACTTYGTGTTCTTCATCATCTTCGGGTCGTTCTTCACCCTCAACCT
IMRSASC06     CTACATGTACCTCTACTTTGTGTTCTTCATCATCTTCGGGTCGTTCTTCACCCTCAACCT
IMRSASD01     CTACATGTACCTCTACTTYGTGTTCTTCATCATCTTCGGGTCGTTCTTCACCCTCAACCT
IMRSASD03     CTACATGTACCTCTACTTCGTGTTCTTCATCATCTTCGGGTCGTTCTTCACCCTCAACCT
IMRSASE02     CTACATGTACCTCTACTTTGTGTTCTTCATCATCTTCGGGTCGTTCTTCACCCTCAACCT
IMRSASE04     CTACATGTACCTCTACTTTGTGTTCTTCATCATCTTCGGGTCGTTCTTCACCCTCAACCT
IMRSASE07     CTACATGTACCTCTACTTYGTGTTCTTCATCATCTTCGGGTCGTTCTTCACYCTCAACCT
IMRSASE09     CTACATGTACCTCTACTTTGTGTTCTTCATCATCTTGGGGTCGTTCTTCACCCTCAACCT
IMRSASF03     CTACATGTACCTCTACTTYGTGTTCTTCATCATCTTCGGGTCGTTCTTCACCCTCAACCT
IMRSASF05     CTACATGTACCTCTACTTYGTGTTCTTCATCATCTTCGGGTCGTTCTTCACYCTCAACCT
IMRSASF08     CTACATGTACCTCTACTTYGTGTTCTTCATCATCTTCGGGTCGTTCTTCACCCTCAACCT
IMRSASF10     CTACATGTACCTCTACTTTGTGTTCTTCATCATCTTSGGGTCGTTCTTCACCCTCAACCT
IMRSASG01     CTACATGTACCTCTACTTCGTGTTCTTCATCATCTTCGGGTCGTTCTTCACCCTCAACCT
IMRSASG04     CTACATGTACCTCTACTTCGTGTTCTTCATCATCTTCGGGTCGTTCTTCACCCTCAACCT
IMRSASG06     CTACATGTACCTCTACTTNGTGTTCTTCATCATCTTCGGGTCGTTCTTCACCCTCAACCT
IMRSASG09     CTACATGTACCTCTACTTCGTGTTCTTCATCATCTTCGGGTCGTTCTTCACCCTYAAYCT
IMRSASH02     CTACATGTACCTCTACTTTGTGTTCTTCATCATCTTCGGGTCGTTCTTCACCCTCAACCT
IMRSASH05     CTACATGTACCTCTACTTYGTGTTCTTCATCATCTTCGGGTCGTTCTTCACCCTCAACCT
IMRSASH07     CTACATGTACCTCTACTTYGTGTTCTTCATCATCTTCGGGTCGTTCTTCACCCTCAACCT
IMRSASH10     CTACATGTACCTCTACTTCGTGTTCTTCATCATCTTCGGGTCGTTCTTCACCCTCAACCT
```

```
                |         70        80        90        100       110       120
IMRSASA01     GTTCATYGGTGTCATCATCGACAACTTCAACGAGCAGAAGAAGAAAGCCGGTGGCTCKCT
IMRSASA10     GTTCATCGGTGTCATCATCGACAACTTCAACGAGCAGAAGAAGAAAGCCGGTGGCTCGCT
IMRSASB05     GTTCATYGGTGTCATCATCGACAACTTCAACGAGCAGAAGAAGAAAGCCGGTGGCTCKCT
IMRSASB07     GTTCATCGGTGTCATCATCGACAACTTCAACGAGCAGAAGAAGAAAGCCGGTGGCTCGCT
IMRSASB08     GTTCATCGGTGTCATYATCGACAACTTCAACGAGCAGAAGAAGAAAGCCGGTGGCTCGCT
IMRSASB10     GTTCATYGGTGTCATCATCGACAACTTCAACGAGCAGAAGAAGAAAGCCGGTGGCTCKCT
IMRSASC01     GTTCATCGGTGTCATCATCGACAACTTCAACGAGCAGAAGAARAAAGCCGGTGGCTCGCT
IMRSASC03     GTTCATCGGTGTCATCATCGACAACTTCAACGAGCAGAAGAAGAAAGCCGGTGGCTCGCT
IMRSASC06     GTTCATYGGTGTCATCATCGACAACTTCAACGAGCAGAAGAAGAAAGCCGGTGGCTCKCT
IMRSASD01     GTTCATCGGTGTCATCATCGACAACTTCAACGAGCAGAAGAAGAAAGCCGGTGGCTCGCT
IMRSASD03     GTTCATCGGTGTCATCATCGACAACTTCAACGAGCAGAAGAAGAAAGCCGGTGGCTCGCT
IMRSASE02     GTTCATCGGTGTCATCATCGACAACTTCAACGAGCAGAAGAAGAAAGCCGGTGGCTCGCT
IMRSASE04     GTTCATYGGTGTCATCATCGACAACTTCAACGAGCAGAAGAAGAAAGCCGGTGGCTCKCT
IMRSASE07     GTTCATCGGTGTCATCATCGACAACTTCAACGAGCAGAAGAAGAAAGCCGGTGGCTCGCT
IMRSASE09     GTTCATCGGTGTCATCATCGACAACTTCAACGAGCAGAAGAAGAAAGCCGGTGGCTCGCT
IMRSASF03     GTTCATCGGTGTCATCATCGACAACTTCAACGAGCAGAAGAAGAAAGCCGGTGGCTCGCT
IMRSASF05     GTTCATCGGTGTCATCATCGACAACTTCAACGAGCAGAAGAAGAAAGCCGGTGGCTCGCT
IMRSASF08     GTTCATCGGTGTCATCATCGACAACTTCAACGAGCAGAAGAAGAAAGCCGGTGGCTCGCT
IMRSASF10     GTTCATCGGTGTCATCATCGACAACTTCAACGAGCAGAAGAAGAAAGCCGGTGGCTCGCT
IMRSASG01     GTTCATCGGTGTCATCATCGACAACTTCAACGAGCAGAAGAAGAAAGCCGGTGGCTCGCT
IMRSASG04     GTTCATCGGTGTCATCATCGACAACTTCAACGAGCAGAAGAAGAAAGCCGGTGGCTCGCT
IMRSASG06     GTTCATCGGTGTCATCATCGACAACTTCAACGAGCAGAAGAAGAAAGCCGGTGGCTCGCT
IMRSASG09     GTTCATCGGTGTCATCATCGACAACTTCAACGAGCAGAAGAAGAAAGCCGGTGGCTCGCT
IMRSASH02     GTTCATCGGTGTCATCATCGACAACTTCAACGAGCAGAAGAAGAAAGCCGGTGGCTCGCT
IMRSASH05     GTTCATCGGTGTCATCATCGACAACTTCAACGAGCAGAAGAAGAAAGCCGGTGGCTCGCT
IMRSASH07     GTTCATCGGTGTCATCATCGACAACTTCAACGAGCAGAAGAAGAAAGCCGGTGGCTCGCT
IMRSASH10     GTTCATCGGTGTCATCATCGACAACTTCAACGAGCAGAAGAAGAAAGCYGGTGGCTCGCT

                |         130       140       150       160       170       180
IMRSASA01     GGAAATGTTCATGACGGAGGATCAGAAAAAGTACTACAACGCAATGAAAAAGATGGGCTC
IMRSASA10     GGAAATGTTCATGACGGAGGATCAGAAAAAGTACTACAACGCAATGAAAAAGATGGGCTC
IMRSASB05     GGAAATGTTCATGACRGAGGATCAGAAAAAGTACTACAACGCAATGAAAAAGATGGGCTC
IMRSASB07     GGAAATGTTCATGACGGAGGATCAGAAAAAGTACTACAACGCAATGAAAAAGATGGGCTC
IMRSASB08     GGAAATGTTCATGACGGAGGATCAGAAAAAGTACTACAACGCAATGAAAAAGATGGGCTC
IMRSASB10     GGAAATGTTCATGACGGAGGATCAGAAAAAGTACTACAACGCAATGAAAAAGATGGGCTC
IMRSASC01     GGAAATGTTCATGACGGAGGATCAGAAAAAGTACTACAACGCAATGAAAAAGATGGGCTC
IMRSASC03     GGAAATGTTCATGACGGAGGATCAGAAAAAGTACTACAACGCAATGAAAAAGATGGGCTC
IMRSASC06     GGAAATGTTCATGACGGAGGATCAGAAAAAGTACTACAACGCAA----------------
IMRSASD01     GGAAATGTTCATGACGGAGGATCAGAAAAAGTACTACAACGCAATGAAAAAGATGGGCTC
IMRSASD03     GGAAATGTTCATGACRGAGGATCAGAAAAAGTACTACAACGCAATGAAAAAGATGGGCTC
IMRSASE02     GGAAATGTTCATGACGGAGGATCAGAAAAAGTACTACAACGCAATGAAAAAGATGGGCTC
IMRSASE04     GGAAATGTTCATGACGGAGGATCAGAAAAAGTACTACAACGCAATGAAAAAGATGGGCTC
IMRSASE07     GGAAATGTTCATGACGGAGGATCAGAAAAAGTACTACAACGCAATGAAAAAGATGGGCTC
IMRSASE09     GGAAATGTTCATGACGGAGGATCAGAAAAAGTACTACAACGCAATGAAA-----------
IMRSASF03     GGAAATGTTCATGACGGAGGATCAGAAAAAGTACTACAACGCAATGAAAAAGATGGGCTC
IMRSASF05     GGAAATGTTCATGACGGAGGATCAGAAAAAGTACTACAACGCAATGAAAAAGATGGGCTC
IMRSASF08     GGAAATGTTCATGACGGAGGATCAGAAAAAGTACTACAACGCAATGAAAAAGATGGGCTC
IMRSASF10     GGAAATGTTCATGACGGAGGATCAGAAAAAGTACTACAACGCAATGA-------------
IMRSASG01     GGAAATGTTCATGACGGAGGATCAGAAAAAGTACTACAACGCAATGAAAAAGATGGGCTC
IMRSASG04     GGAAATGTTCATGACGGAGGATCAGAAAAAGTACTACAACGCAATGAAAAAGATGGGCTC
IMRSASG06     GGAAATGTTCATGACGGAGGATCAGAAAAAGTACTACAACGCAATGAAAAAGATGGGCTC
IMRSASG09     RGAAATGTTCATGACGGAGGATCAGAAAAAGTACTACAACGCAATGAAAAAGATGGGCTC
IMRSASH02     GGAAATGTTCATGACGGAGGATCAGAAAAAGTACTACAACGCAATGAAAAAGATGGGCTC
IMRSASH05     GGAAATGTTCATGACGGAGGATCAGAAAAAGTACTACAACGCAATGAAAAAGATGGGCTC
IMRSASH07     GGAAATGTTCATGACGGAGGATCAGAAAAAGTACTACAACGCAA----------------
IMRSASH10     GGAAATGTTCATGACGGAGGATCAGAAAAAGTACTACAACGCAATGAAAAAGATGGGCTC
```
